# Vemurafenib resistance reprograms melanoma cells towards glutamine dependence

**DOI:** 10.1186/s12967-015-0581-2

**Published:** 2015-07-03

**Authors:** Jenny E Hernandez-Davies, Thai Q Tran, Michael A Reid, Kimberly R Rosales, Xazmin H Lowman, Min Pan, Gatien Moriceau, Ying Yang, Jun Wu, Roger S Lo, Mei Kong

**Affiliations:** Department of Cancer Biology, Beckman Research Institute of City of Hope Cancer Center, Duarte, CA 91010 USA; Animal Tumor Model Program, Division of Comparative Medicine, Beckman Research Institute of City of Hope Cancer Center, Duarte, CA 91010 USA; Division of Dermatology/Department of Medicine, David Geffen School of Medicine, University of California, Los Angeles, CA 90095-1662 USA; Jonsson Comprehensive Cancer Center, David Geffen School of Medicine, University of California, Los Angeles, CA 90095-1662 USA

**Keywords:** Melanoma, Vemurafenib resistance, Glutamine, Cancer metabolism, ^V600^*BRAF*, *NRAS*, BPTES, L-DON

## Abstract

**Background:**

^V600^*BRAF* mutations drive approximately 50% of metastatic melanoma which can be therapeutically targeted by BRAF inhibitors (BRAFi) and, based on resistance mechanisms, the combination of BRAF and MEK inhibitors (BRAFi + MEKi). Although the combination therapy has been shown to provide superior clinical benefits, acquired resistance is still prevalent and limits the overall survival benefits. Recent work has shown that oncogenic changes can lead to alterations in tumor cell metabolism rendering cells addicted to nutrients, such as the amino acid glutamine. Here, we evaluated whether melanoma cells with acquired resistance display glutamine dependence and whether glutamine metabolism can be a potential molecular target to treat resistant cells.

**Methods:**

Isogenic BRAFi sensitive parental ^V600^*BRAF* mutant melanoma cell lines and resistant (derived by chronic treatment with vemurafenib) sub-lines were used to assess differences in the glutamine uptake and sensitivity to glutamine deprivation. To evaluate a broader range of resistance mechanisms, isogenic pairs where the sub-lines were resistant to BRAFi + MEKi were also studied. Since resistant cells demonstrated increased sensitivity to glutamine deficiency, we used glutaminase inhibitors BPTES [bis-2-(5 phenylacetamido-1, 2, 4-thiadiazol-2-yl) ethyl sulfide] and L–L-DON (6-Diazo-5-oxo-l-norleucine) to treat MAPK pathway inhibitor (MAPKi) resistant cell populations both in vitro and in vivo.

**Results:**

We demonstrated that MAPKi-acquired resistant cells uptook greater amounts of glutamine and have increased sensitivity to glutamine deprivation than their MAPKi-sensitive counterparts. In addition, it was found that both BPTES and L-DON were more effective at decreasing cell survival of MAPKi-resistant sub-lines than parental cell populations in vitro. We also showed that mutant *NRAS* was critical for glutamine addiction in mutant *NRAS* driven resistance. When tested in vivo, we found that xenografts derived from resistant cells were more sensitive to BPTES or L-DON treatment than those derived from parental cells.

**Conclusion:**

Our study is a proof-of-concept for the potential of targeting glutamine metabolism as an alternative strategy to suppress acquired MAPKi-resistance in melanoma.

## Background

Melanoma is one of the most aggressive forms of skin cancer affecting an estimated 76,100 individuals per year and accounting for approximately 9,710 deaths in 2014 [1, 2]. According to the American Cancer Society, incidents of melanoma have been increasing steadily for the past 30 years [2]. Oncogenic mutations in the *BRAF* gene, encoding a serine threonine kinase that is an essential part of the RAS–RAF–MEK–ERK signaling cascade have been found in approximately 50–70% of metastatic melanoma [1, 3]. The mutation in *BRAF* is frequently found at residue 600 with valine to glutamic acid (^V600E^*BRAF*) and leads to a hyperactive BRAF kinase which results in uncontrolled cell proliferation and oncogene addiction [1, 4, 5].

Single agent inhibition of the BRAF kinase with small molecule inhibitors such as vemurafenib (PLX4032) and dabrafenib, or double-drug combinations of a BRAF inhibitor with an inhibitor of MEK1/2 such as cobimetinib and trametinib have been successively shown to improve patient survival [6–11]. However, even with the superior efficacy of the double-drug combination, disease control is often cut short by the development of acquired resistance. Genetic resistance mechanisms most commonly result in reactivation of the MAPK pathway through *NRAS* or *KRAS* mutations, ^V600E/K^*BRAF* amplification or alternative splicing [5, 12, 13]. In contrast non-genetic resistance mechanisms often result in MAPK pathway-redundant survival with up-regulated expression of receptor tyrosine kinases such as PDGFRβ [5, 12–14].

It has recently been shown that tumor cell metabolism can be exploited to treat cancer [15]. In the 1920s, Otto Warburg found that cancer cells consume very high rates of glucose and secrete large amounts of lactate in the presence of oxygen, deemed the “Warburg Effect” [15]. This inefficient consumption was designed to meet the biosynthetic and energy production requirements that are frequently seen in tumor cells [16]. It has been shown that in addition to glucose, some cancer cells exhibit “glutamine addiction” to support the anabolic processes that stimulate cell proliferation [17]. Glutamine has been shown to be an essential provider of nitrogen for nucleotide and protein synthesis and affect a critical regulator of protein translation, the mammalian target of rapamycin complex (mTORC)1 [17]. Studies have also pointed at oncogenic changes that allow for regulation of glutamine metabolism in cancer cells. For example oncogenic c-myc has been implicated in the transcriptional regulation of high affinity glutamine transporters to promote glutaminolysis [17]. Pancreatic ductal adenocarcinoma (PDAC) cells have also been shown to be strongly dependent on glutamine and this reprogramming of glutamine metabolism was found to be driven by transcriptional up-regulation of key metabolic enzymes mediated by oncogenic *KRAS* [18]. In melanoma, it has been shown that glutamine transporter ASCT2 was upregulated in ^V600E^*BRAF* mutant melanoma and played a critical role in glutamine uptake and cell proliferation [19]. Therefore, it is highly plausible that disruption of glutamine metabolism can be utilized as a therapeutic approach to treat tumors.

The findings that cancer cells are addicted to glutamine led to therapeutic approaches aimed at impairing glutamine metabolism. Recent work on inhibitors that target glutaminase, the enzyme that catalyzes the conversion of l-glutamine to l-glutamate and ammonia, suggests significant therapeutic potential for cancer treatment. For example, 6-diazo-5-oxo-1-norleucine (L-DON), targets glutaminase on its active site to inhibit tumor growth [20–22]. Another glutaminase inhibitor, bis-2-[5-(phenylacetamido)-1,3,4-thiadiazol-2-yl]ethyl sulfide (BPTES), and its analogs significantly diminish growth of tumor xenografts in vivo and proliferation of cancer cells in vitro for several tumor types, including lymphomas, breast cancers, and gliomas [23–27].

In this study, we demonstrate that melanoma resistant cells uptake glutamine at a higher rate and are more sensitive to glutamine starvation than their vemurafenib sensitive counterparts. Moreover, we show that glutaminase inhibitors BPTES and L-DON can be used to effectively treat resistant cells in vitro and can be used to treat tumors in vivo. We propose targeting glutamine metabolism can be used as an alternative treatment strategy to target tumors resistant to vemurafenib.

## Methods

### Cell culture

Human melanoma parental (vemurafenib sensitive) lines were generated as previously described [4]. Briefly, cells were established directly from patient biopsies and cultured in RPMI 1640 medium with l-glutamine, 10% fetal bovine serum and 1% penicillin, streptomycin, and amphotericin [4]. M229 parental was previously characterized as BRAF^V600E^ homozygous and M249 parental was described as ^V600E^BRAF heterozygous and both equally sensitive to vemurafenib-mediated growth inhibition in vitro and in vivo [4]. Cells were maintained in Dulbecco’s modified Eagle medium (DMEM) with 10% fetal bovine serum (Omega Scientific, Inc) and 4 mM l-glutamine (Omega Scientific, Inc).

Vemurafenib (PLX 4032) single drug resistant (SDR) sub-lines M249 and M229 with ^V600E^*BRAF* positive mutations were generated in vitro by chronic vemurafenib exposure [12]. Briefly, M229 parental line was treated with PLX4032 at 1 mM every 3 days for 4–6 weeks to obtain clonal colonies [12]. PDGFRβ RNA upregulation was found to contribute to M229 resistance [12]. M249 resistant sub-line was derived by successively titrating PLX4032 up to 10 mM [12]. M249 resistant sub-line was shown to harbor a *NRAS*(Q61K) activating mutation not present in the parental M249 cell line that was shown to contribute to resistance [12]. M249 and M229 resistant cells were maintained in DMEM with 10% fetal bovine serum (Omega Scientific, Inc), 4 Mm l-glutamine (Omega Scientific, Inc), and with 1 μM vemurafenib (PLX4032) (Plexxikon).

Double BRAF inhibitor (vemurafenib) and MEK inhibitor (selumetinib) resistant cell lines were generated as previously described [28]. Briefly, the M249 DDR5 double drug resistant cell line (DDR) was generated by treating M249 parental lines with increments of vemurafenib and selumetinib and harbored both the mutant ^V600E^*BRAF* amplification and ^F129L^*MEK1* mutation [28]. M249 double resistant cells were cultured in the above medium maintained with both 1 μM vemurafenib (Plexxikon) and 1 μM selumetinib (Selleck chemicals).

### Nutrient uptake

Parental and single drug resistant cells were seeded at 2 × 10^5^ and 1 × 10^5^ cells/well, respectively in 6 well plates in triplicate and allowed to incubate overnight. Media only was also plated as a control for parental and resistant cells. At 24 and 48 h upon cells reaching 60% confluence, cell medium was collected and transferred to micro-centrifuge tubes and placed in the Nova Bioprofiler 100plus Analyzer (Nova Biomedical) for measurement of nutrient uptake. In addition, cells were counted using the Biorad TC20 automated cell counter. Upon measurement, medium only control values were subtracted from readings and values/cell were calculated.

### DIMSCAN cell in vitro cytotoxicity assays

M249 and M229 parental, single drug resistant (SDR) cells, and M249 DDR5 double drug resistant cells (DDR) were seeded at 3,000 cells/well and 2,000 cells/well (respectively) in 96 well plates. Cells were allowed to settle overnight prior to treatment. After overnight incubation, cells were washed with 1× PBS to remove traces of medium. Parental cells were treated with either media with or without l-glutamine or d-glucose, 10 μM BPTES [bis-2-(5 phenylacetamido-1, 2, 4-thiadiazol-2-yl) ethyl sulfide] (LT Pharma, Inc.) or 10 μM L-DON (6-Diazo-5-oxo-l-norleucine) (Sigma-Aldrich) alone. Single drug resistant cells were treated in combination with 1 μM vemurafenib. Double drug resistant cells were treated in combination with both 1 μM vemurafenib and 1 μM selumetinib. BPTES stock solution was dissolved in DMSO to a final working concentration of 10 mM and stored at −20°C. L-DON was dissolved in water to a final concentration of 100 mM and also stored at −20°C. DMSO and water only controls were added to each well. Cells were also treated with media without glutamine or glucose containing dialyzed fetal bovine serum (Gemini Scientific). Treated cells were incubated for 24, 48, and 72 h. To prepare for DIMSCAN analysis, 0.5% Eosin Y was added to spaces between wells on a 96 well plate. A solution of fluorescein diacetate (FDA) at 10 mg/ml was added to 0.5% Eosin Y solution to make a working concentration of 40μg/ml to add to cells. After a 20 min incubation, cells were scanned for fluorescence using DIMSCAN, a fluorescence-based digital image microscopy system [29]. Results were analyzed to obtain survival percentage using the DIMSCAN data analyzer by DTT (Children’s Hospital Los Angeles).

### Western blots

M249 and M229 parental and single drug resistant cells were seeded in 6 cm plates overnight to obtain 60% confluence the following day. Cells were washed with 1× PBS. Medium with or without glutamine was added to cells. After 24 h, cells were lysed with RIPA buffer and protease inhibitors and lysates were run on SDS page gels. Gels were transferred to a nitrocellulose membrane and probed with anti cleaved-PARP (Cell Signaling Technology) to assess apoptosis.

### AnnexinV/DAPI staining

M249 and M229 cells were plated at 5 × 10^4^ (parental) and 4 × 10^4^ cells (resistant) in 12 well plates. Double drug resistant lines were plated at 4 × 10^4^ cells in 12 well plates. After overnight incubation and reaching 60% confluence, cells were stained with flourochrome-conjugated Annexin V and propidium iodide using eBioscience reagents and Annexin V staining protocol. Cell acquisition was completed using the 9-color CyAn ADP from Beckman Coulter (Miami, FL, USA). Research reported in this publication included work performed in the Analytical Cytometry Core supported by the National Cancer Institute of the National Institutes of Health under award number P30CA33572. Cells were analyzed using FlowJo data analysis software (Ashland, OR, USA).

### shRNA knockdown

shRNA lentiviral particles (Sigma) were used to infect M249 single drug resistant cells at 50% confluence using polybrene (Hexadimethrine bromide) (Sigma). Cells were selected for puromycin resistance (Sigma) after 48 h. Bulk cell populations were utilized for experiments. Knockdown efficiency was assessed using quantitative PCR and determination of relative mRNA expression of *NRAS*.

### In vivo xenograft model

All animal studies were performed according to approved IACUC protocols at the City of Hope Cancer Center. Nod Scid Gamma (NSG) mice were injected with 5 × 10^5^ of M249 parental or single drug resistant cells subcutaneously on the right flank. When tumor size reached an average of 100 mm^3^ tumor cell volume, mice were treated with 15 mg/kg of BPTES or vehicle control (DMSO) every other day through intraperitoneal injection. Measurements were taken for tumor length and width. Tumor volume (mm^3^) was calculated by multiplying (length × width × width)/2. NCr nude mice (Taconic) were injected with 2 × 10^6^ of M249 single drug resistant cells subcutaneously on the right flank. When tumor size reached an average of 100 mm^3^, they were treated bi-weekly with 20 mg/kg of L-DON or vehicle control (water).

### Statistical analysis

All in vitro experiments were performed in triplicate and repeated a minimum of three times. In the figures, representative experiments are shown. Paired t tests were done to calculate p values for representative experiments using Graphpad software (San Diego, CA, USA). Values under p < 0.05 were considered significant.

## Results

### Vemurafenib resistant cells uptake and use glutamine at a higher rate than vemurafenib sensitive cells

**Figure 1:**
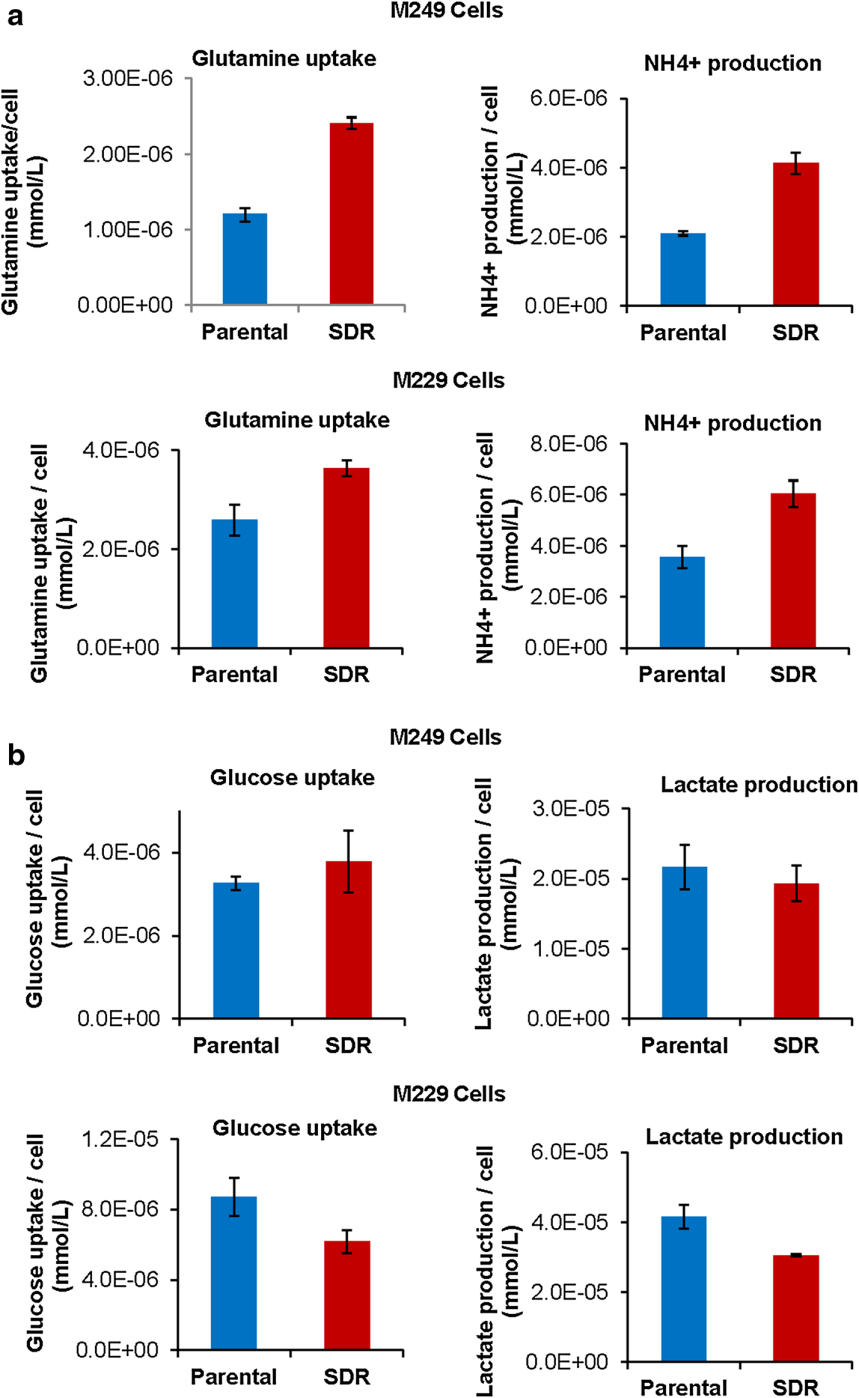
Vemurafenib resistant cells uptake and use glutamine at a higher rate than vemurafenib sensitive cells. Melanoma M249 and M229 vemurafenib sensitive (parental) and single drug resistant (SDR) cell lines were plated to be 60% confluent and medium was changed after overnight incubation. Medium only was used as a control. Cells were then cultured for 24 h. Medium was then taken from growing cells and measurements were obtained of glutamine uptake, NH_4_^+^ production, glucose uptake, and lactate production using the Nova Bioprofiler 100 plus as described in materials and methods. Cells were counted and results are expressed as **a** glutamine uptake per cell (mmol/L) and NH_4_^+^ production per cell (mmol/L) and **b** glucose uptake per cell (mmol/L) and lactate production per cell (mmol/L) and are representative of the average (±standard deviation) of triplicate experiments.

We first aimed to assess whether there were any differences in nutrient uptake between isogenic BRAFi sensitive parental ^V600^*BRAF* mutant melanoma cell lines and single drug resistant (derived by chronic treatment with vemurafenib) sub-lines. We found that both M249 and M229 single drug resistant (SDR) cells had greater uptake of glutamine than their parental counterparts (Figure 1a). It is also known that glutaminase activity generates free ammonia, therefore, we also tested for NH4^+^ production [15]. We found that both M249 and M229 single drug resistant cells had greater amounts of ammonia production than parental cell populations, which is indicative of higher glutamine usage (Figure 1a). To exclude the possibility that the resistant cells display a general increase in metabolism, we also measured glucose uptake and lactate production in M249 and M229 parental and single drug resistant cells (Figure 1b). In contrast to the glutamine uptake, there were no significant differences in glucose uptake and lactate production between parental and single drug resistant cells (Figure 1b). These data suggest that single drug resistant cells may be more dependent on glutamine for growth and proliferation than vemurafenib-sensitive (parental) cell populations.

### Vemurafenib resistant cells are more sensitive to glutamine deprivation

**Figure 2:**
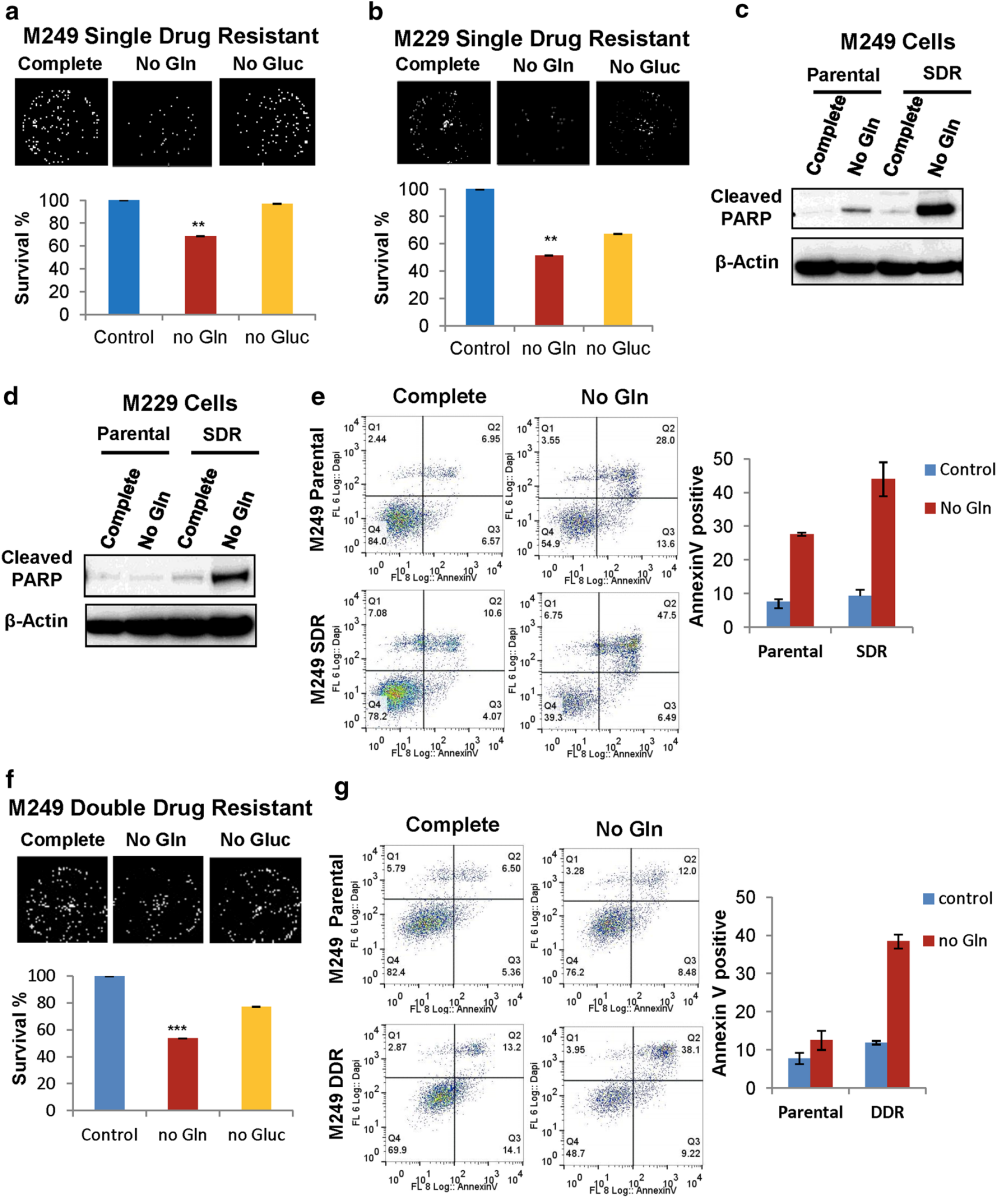
Vemurafenib resistant cells are more sensitive to glutamine deprivation. **a** Melanoma M249 and **b** M229 single drug resistant (SDR) cells were cultured in the presence of medium with or without glutamine (Gln) or glucose (Gluc). After 24 h representative images and surviving fractions (survival %) compared to control cells in complete medium are shown using DIMSCAN, a microcomputer fluorescence-based cytotoxicity assay (n = 6, **p < 0.01). Western blot analysis was also used to assess levels of cleaved PARP as an indicator of apoptosis in both **c** M249 and **d** M229 parental and SDR cell lines. **e** Apoptosis was also assessed using flow cytometry of M249 parental and resistance cells stained with AnnexinV and DAPI. M249 parental and SDR cells were cultured in the presence of medium with and without glutamine for 24 h. Representative *dot blots* and percentages of AnnexinV positive cells are shown. **f** Melanoma double drug resistant (DDR) cell line M249 DDR5 was cultured in the presence of medium with and without glutamine (Gln) or glucose (Gluc). After 24 h representative images and surviving fractions (survival %) compared to control cells in complete medium are shown using DIMSCAN, a microcomputer fluorescence-based cytotoxicity assay (n = 6, ***p < 0.001). **g** Apoptosis was also assessed using flow cytometry of M249 parental and DDR cells stained with AnnexinV and DAPI. M249 parental and DDR cells were cultured in the presence of medium with and without glutamine for 24 h. Representative *dot blots* and percentages of AnnexinV positive cells are shown.

In order to test this, we aimed to deprive M249 and M229 single drug resistant cells of glutamine and glucose to observe the effects on cell survival. We found that both M249 and M229 single drug resistant (SDR) populations were more sensitive to glutamine deprivation than glucose deprivation (Figure 2a, b). In addition, both parental and single drug resistant cells were deprived of glutamine and tested for apoptosis by measuring PARP cleavage using Western blot analysis. M249 and M229 single drug resistant cells both had higher levels of PARP cleavage upon glutamine deprivation than parental counterparts indicating increased levels of apoptosis (Figure 2c, d). In correlation with the Western blot results, there were more apoptotic cells upon glutamine deprivation in the M249 single drug resistant line than the parental line measured by Annexin V staining (Figure 2e). To further evaluate a broader range of resistance mechanisms, melanoma isogenic sub-lines with acquired vemurafenib and MEK inhibitor double drug resistance (DDR) were also used. We tested the double drug resistant (DDR) M249 cell line containing both *BRAF* amplication and *MEK1* mutations for sensitivity to glutamine. Similar to the single drug resistant lines, the M249 double drug resistant line was found to be more sensitive to glutamine deprivation than glucose deprivation (Figure 2f). In addition, we tested for apoptosis via Annexin V staining upon glutamine deprivation and found that M249 double drug resistant cells were also more sensitive to glutamine deprivation than parental counterparts (Figure 2g). These results highly suggest that resistant cells are more dependent on glutamine for their survival, and that targeting glutamine metabolism with inhibitors aimed at blocking glutamine usage may be a way to specifically kill the vemurafenib resistant cells.

### Vemurafenib resistant cells are more sensitive to glutaminase inhibitors

**Figure 3:**
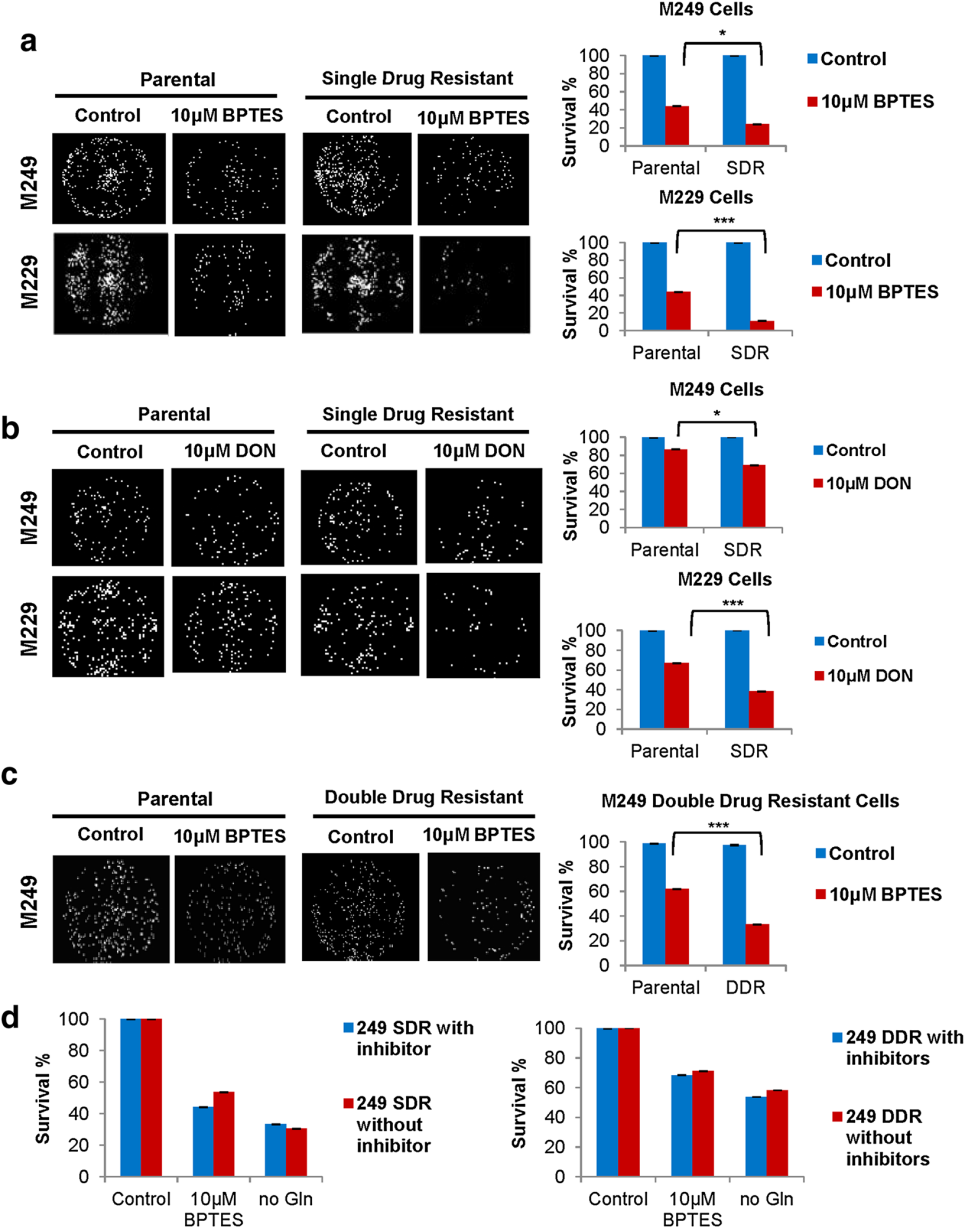
Vemurafenib resistant cells are more sensitive to glutaminase inhibitors. **a** M249 and M229 parental single drug resistant (SDR) cells were cultured in the presence of medium control (DMSO) or 10 μM BPTES for 48 h. SDR cell lines were cultured in combination of vemurafenib (1 μM) and BPTES. Surviving cell fraction percentage (survival %) compared to vehicle control (DMSO) was assessed using DIMSCAN technology (n = 6, ***p < 0.001, *p < 0.05). **b** M249 and M229 parental and single drug resistant cells were cultured in the presence of vehicle control (water) or 10 μM L-DON for 48 h. SDR cell lines received a combination of vemurafenib (1 μM) and L-DON. Surviving cell fraction percentage (survival %) compared to vehicle control (DMSO) was assessed using DIMSCAN technology (n = 6, ***p < 0.001, *p < 0.05). **c** M249 parental and double drug resistant (DDR) cells were cultured in the presence of medium control (DMSO) or 10 μM BPTES for 48 h. DDR cell lines were cultured in combination with vemurafenib (1 μM), selumetinib (1 μM), and BPTES. Surviving cell fraction percentage compared to vehicle control (DMSO) (survival %) was assessed using DIMSCAN technology (n = 6, ***p < 0.001). **d** M249 single (SDR) and double drug (DDR) resistant lines were cultured in the presence or absence of inhibitor (1 μM vemurafenib for SDR and 1 μM vemurafenib and 1 μM selumentib for DDR) and subsequently treated with 10 μM BPTES or deprived of glutamine (Gln) for 48 h. Surviving cell fraction percentage compared to vehicle control (DMSO) (survival %) was assessed using DIMSCAN technology.

To test whether we can target glutamine metabolism to treat resistant cells, we used glutaminase inhibitors, BPTES and L-DON to treat both parental and resistant cell populations. We then assessed cell survival upon treatment and observed whether resistant cell populations were in fact more sensitive to glutamine uptake. We found that, although both parental and single drug resistant populations were sensitive to glutaminase inhibitors BPTES and L-DON, single drug resistant cells in combination with vemurafenib were much more sensitive as demonstrated by reduced cell survival percentages (Figure 3a, b). To further examine whether double drug resistant cell lines were also sensitive to glutaminase inhibitors, we tested M249 double drug resistant cell line for survival after treatment with BPTES. We found that the double drug resistant line (similar to the single drug resistant line) was more sensitive to BPTES than parental counterparts (Figure 3c). To address whether this effect required BRAF inhibition or BRAF/MEK1 inhibition, we treated both M249 single and double drug resistant cell lines with BPTES and without glutamine in the presence or absence of BRAF/MEK inhibitors. We found that treatment of single or double drug resistant cell lines does not require the presence of the inhibitor to become sensitive to glutamine deprivation or BPTES treatment (Figure 3d). These results suggest that glutaminase inhibitors may be used as a strategy to target resistant cell populations.

### Knock down of *NRAS* in vemurafenib resistant cells reduces sensitivity to glutaminase inhibitor

**Figure 4:**
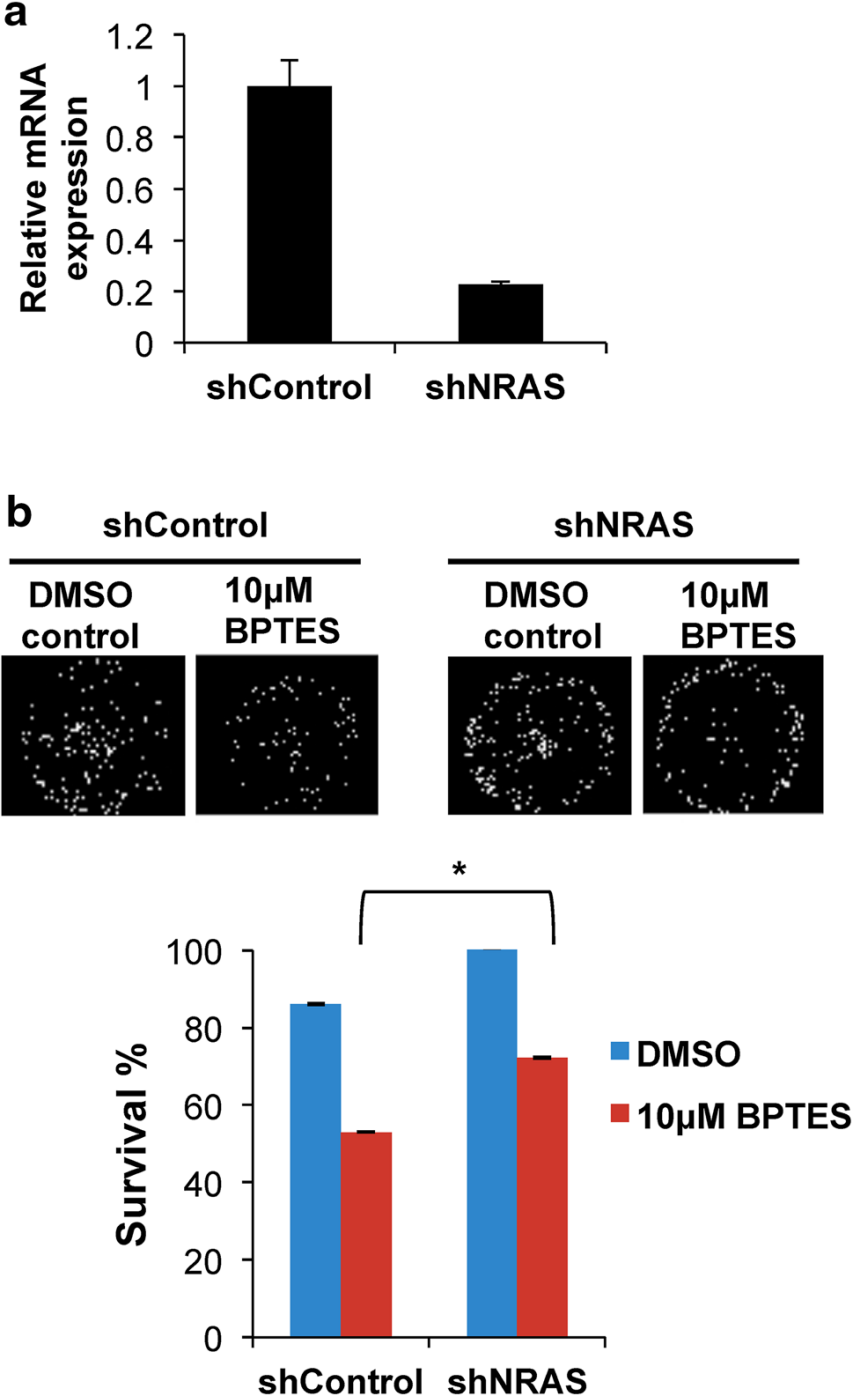
Knock down of *NRAS* in vemurafenib resistant cells reduces sensitivity to glutaminase inhibitor. Melanoma M249 resistant cells were infected with shNRAS or shControl lentiviral particles and selected for puromycin resistance after 48 h. **a** Quantitative PCR was used to measure relative mRNA expression of NRAS in shControl and shNRAS infected cells. **b** shNRAS or shControl cells were cultured in the presence of 10 μM BPTES for 48 h or with vehicle control (DMSO). Surviving cell fraction percentage (survival %) compared to vehicle control (DMSO) was determined using DIMSCAN analysis (n = 6, *p < 0.05).

To determine whether resistance acquired *NRAS* mutations had a role in the transformation of M249 resistant cells to glutamine dependence, cells with stable knockdown of *NRAS* and scrambled shRNA controls were prepared using short hairpin RNA (shRNA). First, we assessed whether the shRNA was successful at reducing levels of *NRAS* in the cell. Indeed, relative mRNA expression of *NRAS* was reduced in shNRAS knockdown cells well below control levels (Figure 4a). Control and shNRAS knock down cells were subsequently treated with BPTES and assessed for cell survival without the presence of BRAF inhibitor vemurafenib in the medium. We found that knocking down *NRAS* allowed cells to become less sensitized to the glutaminase inhibitor BPTES when compared to control cell lines (Figure 4b). These results suggest that resistance acquired mutations in *NRAS* contribute to reprogramming of resistant cells to glutamine dependence.

### Vemurafenib resistant melanoma tumors are sensitive to glutaminase inhibitor treatment in vivo

**Figure 5:**
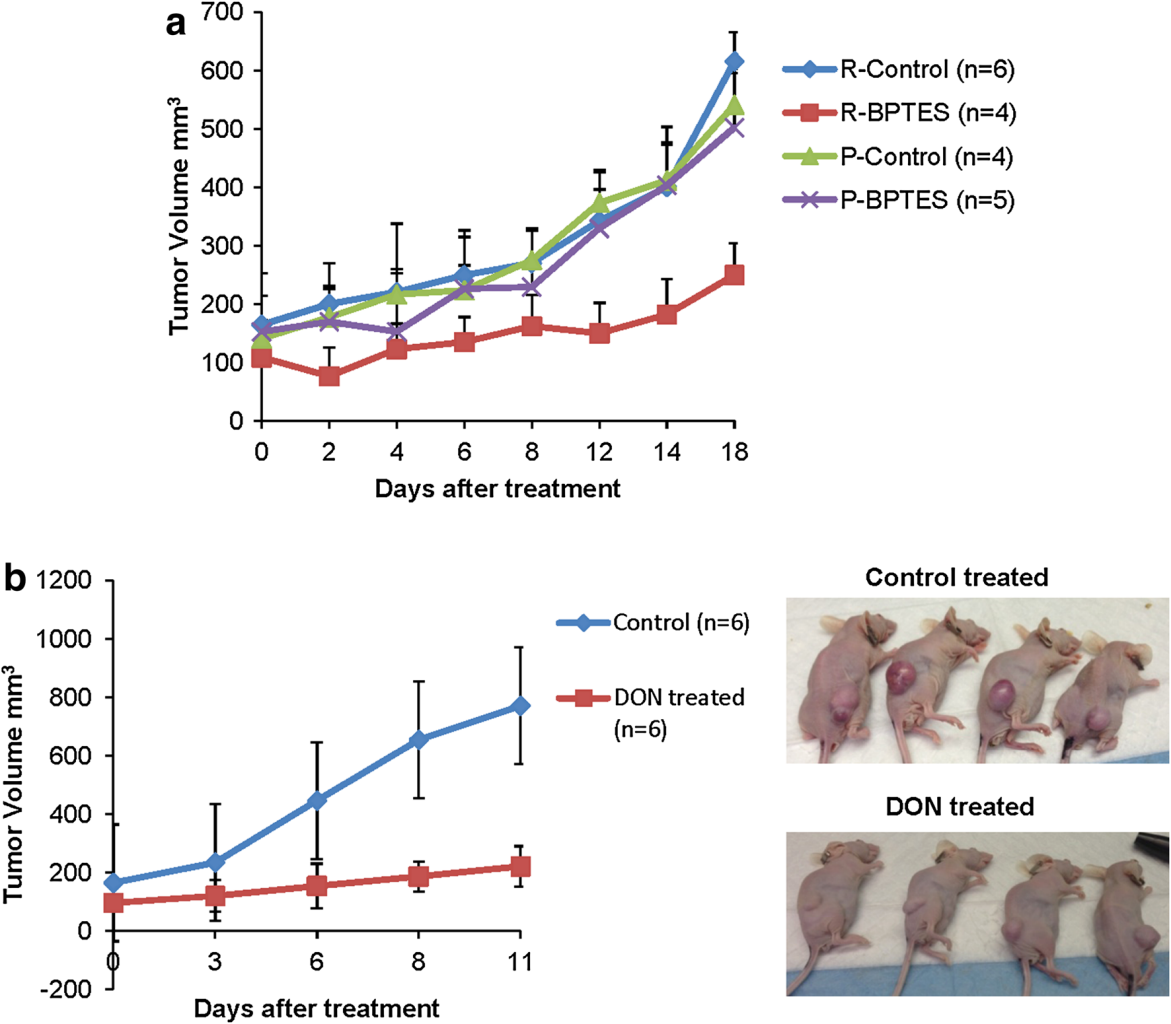
Vemurafenib resistant melanoma tumors are sensitive to glutaminase inhibitor treatment in vivo. **a** Nod Scid Gamma (NSG) mice were injected with 5 × 10^6^ of M249 parental or single drug resistant cells subcutaneously on the right flank. When tumor size reached an average of 100 mm^3^ tumor cell volume, treatment with 15 mg/kg of BPTES or vehicle control (DMSO) was provided every other day through intraperitoneal injection. Measurements were taken for tumor length and width. Tumor volume (mm^3^) was calculated by multiplying (length × width × width)/2. *Graph* represents mean tumor volume ± SD. **b** Nude mice were injected with 2 × 10^6^ of M249 resistant cells subcutaneously on right flank. When tumor size reached an average of 100 mm^3^ tumor cell volume, treatment with 20 mg/kg of L-DON or vehicle control (water) was provided twice a week through intraperitoneal injection. *Graph* represents mean tumor volume ± SD.

Since in vitro data demonstrated the potential of glutaminase inhibitors to treat resistant cell lines, we asked whether these inhibitors would also be effective in vivo. To do this, xenograft experiments with NSG mice were used to inject mice with both M249 parental (vemurafenib sensitive) and M249 single drug resistant cell lines and subsequently treat with glutaminase inhibitor BPTES. As shown in Figure 5a, BPTES dramatically suppressed tumor growth in mice injected with M249 single drug resistant cells compared to mice injected with the parental (sensitive) cells. As the BPTES treatment displayed minimal effect on tumor growth from parental cells, we next focused to validate whether targeting glutamine metabolism is efficient to inhibit resistant tumor growth using a different glutaminase inhibitor, L-DON. Similarly, we found that L-DON effectively blocked vemurafenib resistant tumor growth (Figure 5b).

## Discussion

Here, we demonstrated that M249 and M229 vemurafenib resistant cells have been reprogrammed to become increasingly dependent on glutamine when compared to their vemurafenib-sensitive counterparts. In addition, we show that acquisition of resistance increased sensitivity of these cells to glutamine deprivation. We also demonstrated that, in addition to single drug resistant lines, double drug resistant lines harboring both the *BRAF* amplification and *MEK1* mutations became sensitive to glutamine deprivation. We were successfully able to exploit this sensitivity to glutamine to kill these single and double drug resistant cells with a combination of glutaminase inhibitors BPTES or L-DON and vemurafenib. In both single and double drug resistant cases, decreased cell survival was observed. In addition, knocking down *NRAS* in the M249 resistant cells decreased the sensitivity of these cells to both glutaminase inhibitors, indicating the possibility that resistance acquired mutations may influence this increased dependence on glutamine. These results were also obtained in vivo as treating mice injected with M249 single drug resistant cells with BPTES or L-DON resulted in a significant decrease of tumor volume.

Single agent treatment with BRAF inhibitors such as vemurafenib and dabrafenib have demonstrated improved survival for patients with ^V600E^*BRAF* mutant melanoma and are currently approved by the US Food and Drug Administration for treatment [13]. In addition, combination therapy of BRAF inhibitors with allosteric MEK1 and MEK2 inhibitors are also in clinical trials and have been approved for treatment for *BRAF* mutant melanomas [7, 13, 28, 30, 31]. However, as with single agent therapies, combination therapy with BRAF and MEK inhibitors have also led to the development of mechanisms of resistance by further amplifying existing resistance mutations or by reactivating the MAPK pathway [9, 13, 28]. These resistance mechanisms have led to the need for alternative treatment options [13]. Recently, blocking the immune-regulatory checkpoints that limit T cell responses using antagonistic antibodies against the programmed death 1 pathway (PD-1) and one of its ligands, programmed death ligand 1 (PD-L1), and blockade of CTLA-4/B7 (cytotoxic T lymphocyte-associated antigen-4) interaction with anti-CTLA-4 antagonistic monoclonal antibodies (mAbs) have demonstrated high clinical benefits in melanoma patients [32, 33]. Besides targeting immuno-responses, whether targeting altered metabolism to treat resistant melanoma cells has not yet been explored. Our study suggests that inhibition of glutamine metabolism could be a promising way to treat single and double resistant tumor types.

As our data suggested that different resistance models could all lead to “glutamine addiction”, it is critical to identify how these cells were able to reprogram to a preferential glutamine dependent metabolism. It will be important in the future to look at glutamine transporters or metabolic enzymes involved in the glutamine metabolic pathway to assess how mutations are directly involved in modifying the intrinsic metabolism of these cells. This will allow for the development of more targeted therapies that can be used to target tumors that do become glutamine dependent.

Overall, these data suggest that both vemurafenib single drug resistant and vemurafenib/selumetinib (MEK inhibitor) double drug resistant lines are sensitive to glutamine and to glutaminase inhibitors. Therefore targeting glutamine metabolism may be a useful tool in the future to treat vemurafenib resistant melanoma.

## Conclusion

Currently, therapy used for the treatment of vemurafenib resistant melanoma involves combination BRAF and MEK inhibitors to target inhibition of the MAPK pathway. Due to the aggressiveness of melanoma and its unique ability to develop resistant mutations even after treatment with combination of BRAF and MEK inhibitors, it will become necessary to develop alternative forms of therapy to evade resistance. Our study demonstrated that targeting glutamine metabolism can be a way to potentially treat vemurafenib resistant melanoma.

## References

[1] Bucheit AD, Davies MA (2014). Emerging insights into resistance to BRAF inhibitors in melanoma. Biochem Pharmacol.

[2] American Cancer Society FaF (2014) Facts and figures 2014

[3] Davies H, Bignell GR, Cox C, Stephens P, Edkins S, Clegg S (2002). Mutations of the BRAF gene in human cancer. Nature.

[4] Sondergaard JN, Nazarian R, Wang Q, Guo D, Hsueh T, Mok S (2010). Differential sensitivity of melanoma cell lines with BRAFV600E mutation to the specific Raf inhibitor PLX4032. J Transl Med.

[5] Shi H, Kong X, Ribas A, Lo RS (2011). Combinatorial treatments that overcome PDGFRbeta-driven resistance of melanoma cells to V600EB-RAF inhibition. Cancer Res.

[6] McArthur GA, Chapman PB, Robert C, Larkin J, Haanen JB, Dummer R (2014). Safety and efficacy of vemurafenib in BRAF(V600E) and BRAF(V600K) mutation-positive melanoma (BRIM-3): extended follow-up of a phase 3, randomised, open-label study. Lancet Oncol.

[7] Hauschild A, Grob JJ, Demidov LV, Jouary T, Gutzmer R, Millward M (2012). Dabrafenib in BRAF-mutated metastatic melanoma: a multicentre, open-label, phase 3 randomised controlled trial. Lancet.

[8] Ribas A, Gonzalez R, Pavlick A, Hamid O, Gajewski TF, Daud A (2014). Combination of vemurafenib and cobimetinib in patients with advanced BRAF(V600)-mutated melanoma: a phase 1b study. Lancet Oncol.

[9] Queirolo P, Picasso V, Spagnolo F (2015). Combined BRAF and MEK inhibition for the treatment of BRAF-mutated metastatic melanoma. Cancer Treat Rev.

[10] Robert C, Karaszewska B, Schachter J, Rutkowski P, Mackiewicz A, Stroiakovski D (2015). Improved overall survival in melanoma with combined dabrafenib and trametinib. N Engl J Med.

[11] Flaherty KT, Infante JR, Daud A, Gonzalez R, Kefford RF, Sosman J (2012). Combined BRAF and MEK inhibition in melanoma with BRAF V600 mutations. N Engl J Med.

[12] Nazarian R, Shi H, Wang Q, Kong X, Koya RC, Lee H (2010). Melanomas acquire resistance to B-RAF(V600E) inhibition by RTK or N-RAS upregulation. Nature.

[13] Spagnolo F, Ghiorzo P, Orgiano L, Pastorino L, Picasso V, Tornari E (2015). BRAF-mutant melanoma: treatment approaches, resistance mechanisms, and diagnostic strategies. Onco Targets Therapy.

[14] Shi H, Hugo W, Kong X, Hong A, Koya RC, Moriceau G (2014). Acquired resistance and clonal evolution in melanoma during BRAF inhibitor therapy. Cancer Discov.

[15] DeBerardinis RJ, Cheng T (2010). Q’s next: the diverse functions of glutamine in metabolism, cell biology and cancer. Oncogene.

[16] Parmenter TJ, Kleinschmidt M, Kinross KM, Bond ST, Li J, Kaadige MR (2014). Response of BRAF-mutant melanoma to BRAF inhibition is mediated by a network of transcriptional regulators of glycolysis. Cancer Discov.

[17] Wise DR, Thompson CB (2010). Glutamine addiction: a new therapeutic target in cancer. Trends Biochem Sci.

[18] Son J, Lyssiotis CA, Ying H, Wang X, Hua S, Ligorio M (2013). Glutamine supports pancreatic cancer growth through a KRAS-regulated metabolic pathway. Nature.

[19] Wang Q, Beaumont KA, Otte NJ, Font J, Bailey CG, van Geldermalsen M (2014). Targeting glutamine transport to suppress melanoma cell growth. Int J Cancer J Int du Cancer.

[20] Kisner DL, Catane R, Muggia FM (1980). The rediscovery of DON (6-diazo-5-oxo-l-norleucine). Recent Results Cancer Res.

[21] Sklaroff RB, Casper ES, Magill GB, Young CW (1980). Phase I study of 6-diazo-5-oxo-l-norleucine (DON). Cancer Treat Rep.

[22] Thangavelu K, Chong QY, Low BC, Sivaraman J (2014). Structural basis for the active site inhibition mechanism of human kidney-type glutaminase (KGA). Sci Rep.

[23] Shukla K, Ferraris DV, Thomas AG, Stathis M, Duvall B, Delahanty G (2012). Design, synthesis, and pharmacological evaluation of bis-2-(5-phenylacetamido-1,2,4-thiadiazol-2-yl)ethyl sulfide 3 (BPTES) analogs as glutaminase inhibitors. J Med Chem.

[24] Thangavelu K, Pan CQ, Karlberg T, Balaji G, Uttamchandani M, Suresh V (2012). Structural basis for the allosteric inhibitory mechanism of human kidney-type glutaminase (KGA) and its regulation by Raf–Mek–Erk signaling in cancer cell metabolism. Proc Natl Acad Sci USA.

[25] Le A, Lane AN, Hamaker M, Bose S, Gouw A, Barbi J (2012). Glucose-independent glutamine metabolism via TCA cycling for proliferation and survival in B cells. Cell Metab.

[26] Seltzer MJ, Bennett BD, Joshi AD, Gao P, Thomas AG, Ferraris DV (2010). Inhibition of glutaminase preferentially slows growth of glioma cells with mutant IDH1. Cancer Res.

[27] Robinson MM, McBryant SJ, Tsukamoto T, Rojas C, Ferraris DV, Hamilton SK (2007). Novel mechanism of inhibition of rat kidney-type glutaminase by bis-2-(5-phenylacetamido-1,2,4-thiadiazol-2-yl)ethyl sulfide (BPTES). Biochem J.

[28] Moriceau G, Hugo W, Hong A, Shi H, Kong X, Yu CC (2015). Tunable-combinatorial mechanisms of acquired resistance limit the efficacy of BRAF/MEK cotargeting but result in melanoma drug addiction. Cancer Cell.

[29] Keshelava N, Frgala T, Krejsa J, Kalous O, Reynolds CP (2005). DIMSCAN: a microcomputer fluorescence-based cytotoxicity assay for preclinical testing of combination chemotherapy. Methods Mol Med.

[30] Chapman PB, Hauschild A, Robert C, Haanen JB, Ascierto P, Larkin J (2011). Improved survival with vemurafenib in melanoma with BRAF V600E mutation. N Engl J Med.

[31] Flaherty KT, Robert C, Hersey P, Nathan P, Garbe C, Milhem M (2012). Improved survival with MEK inhibition in BRAF-mutated melanoma. N Engl J Med.

[32] Homet Moreno B, Parisi G, Robert L, Ribas A (2015). Anti-PD-1 therapy in melanoma. Semin Oncol.

[33] Queirolo P, Morabito A, Laurent S, Lastraioli S, Piccioli P, Ascierto PA (2013). Association of CTLA-4 polymorphisms with improved overall survival in melanoma patients treated with CTLA-4 blockade: a pilot study. Cancer Invest.

